# Supplementary material for: Influence of Parameters on the Death Pathway of Gastric Cells Induced by Gold Nanosphere Mediated Phototherapy
Source: Nanomaterials (Basel). 2022 Feb 15;12(4):646. doi: 10.3390/nano12040646 (PMC8878397; doi:10.3390/nano12040646)
Supplement: Supplementary file 1 [file nanomaterials-12-00646-s001.zip › nanomaterials-1574040-supplementary.pdf]

Supplementary Materials

Influence of parameters on the death pathway of gastric cells induced by gold nanosphere mediated phototherapy

Jing Xin, Lei Fu, Jing Wang, Sijia Wang, Luwei Zhang, Zhenxi Zhang*, Cuiping Yao*

Xi'an Jiaotong University, School of Life Science and Technology, Institute of Biomedical Photonics and Sensing, Key Laboratory of Biomedical Information Engineering of Ministry of Education, Xi'an, Shaanxi, China

* Correspondence: Tel: 82663286-83; Fax 82663286-83

E-mail addresses: zsycp@mail.xjtu.edu.cn

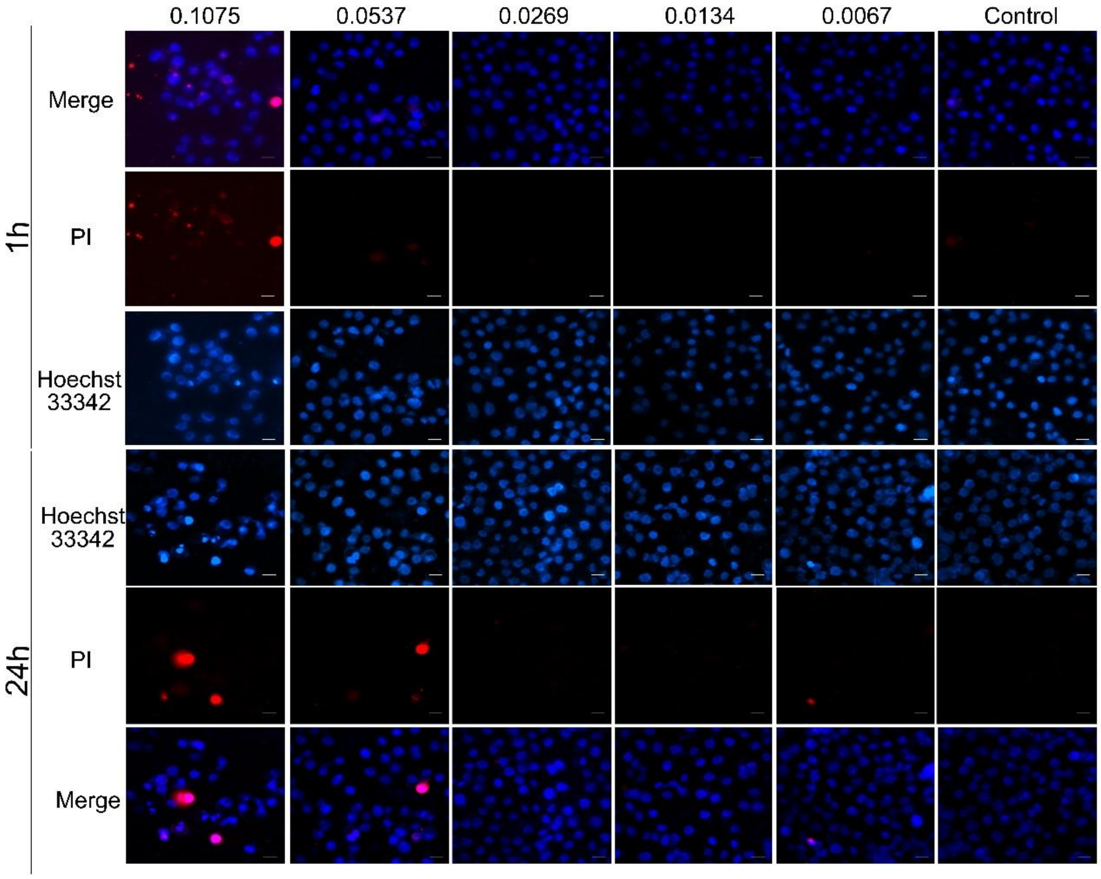

Figure S1. Apoptosis and necrosis induced by PEG-AuS irradiated with nanosecond laser at 4 h incubation time and 11.47 mJ/cm^2 irradiation dosage.

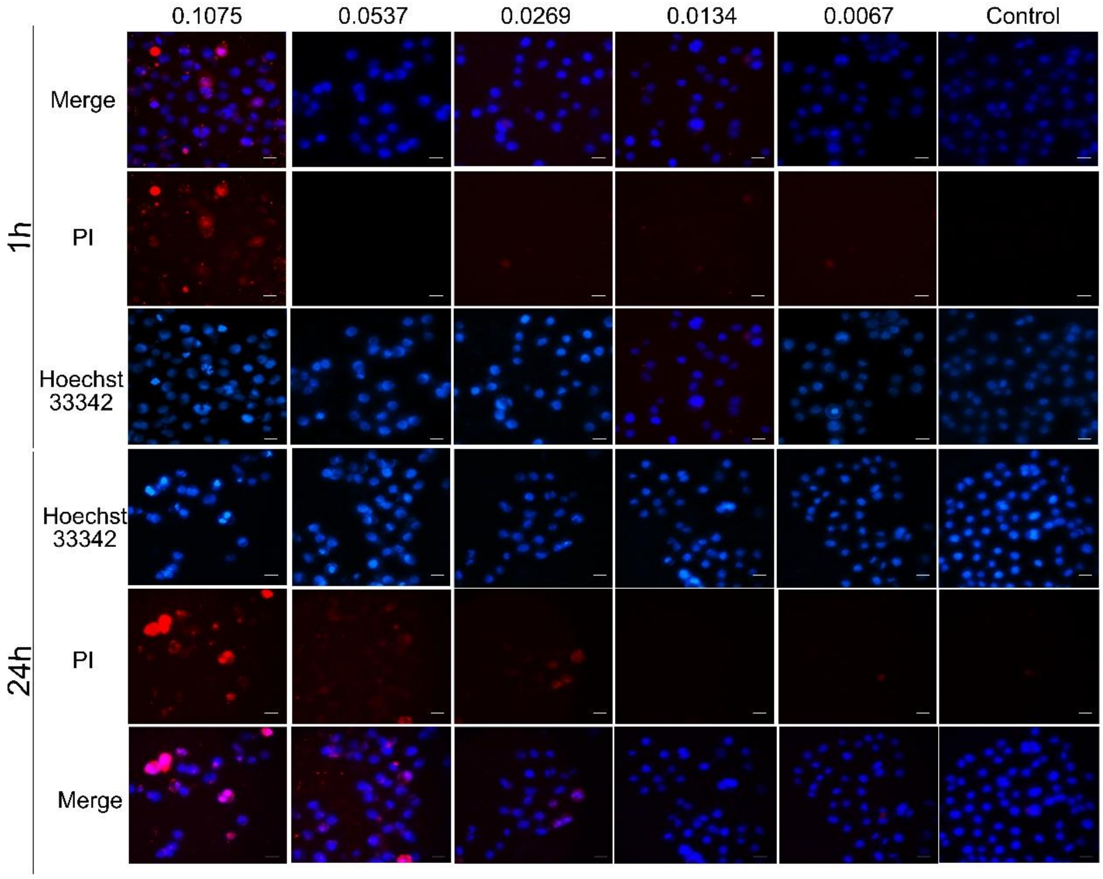

Figure S2. Apoptosis and necrosis induced by PEG-AuS irradiated with nanosecond laser at 4 h incubation time and 22.93 mJ/cm^2 irradiation dosage.

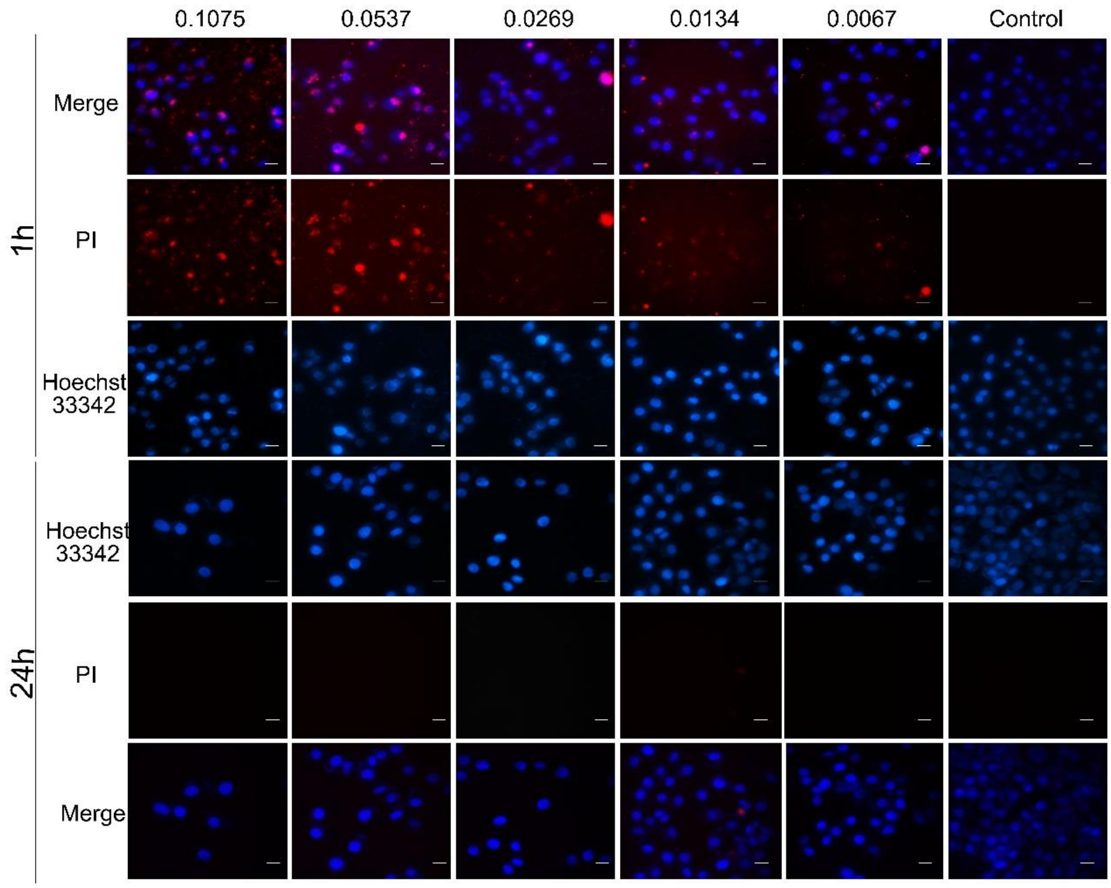

Figure S3. Apoptosis and necrosis induced by PEG-AuS irradiated with nanosecond laser at 6 h incubation time and 11.47 mJ/cm^2 irradiation dosage.

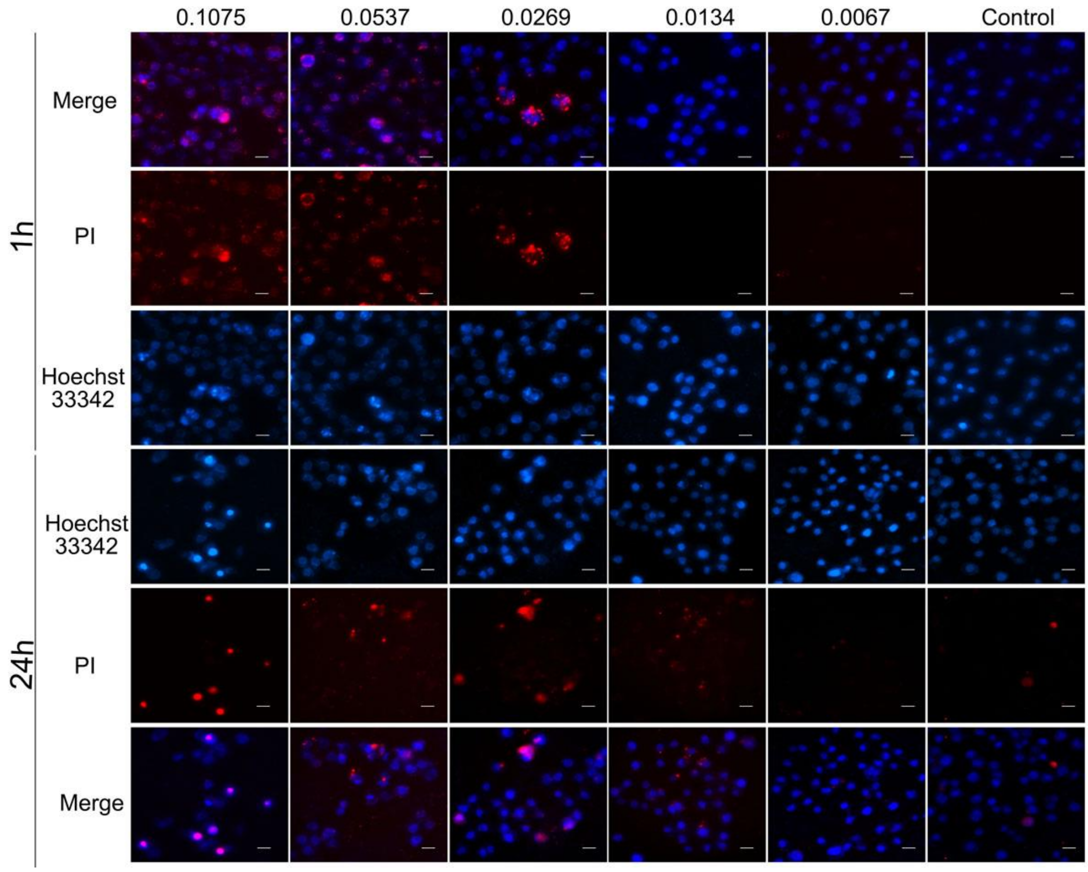

Figure S4. Apoptosis and necrosis induced by PEG-AuS irradiated with nanosecond laser at 6h incubation time and 22.93 mJ/cm^2 irradiation dosage.

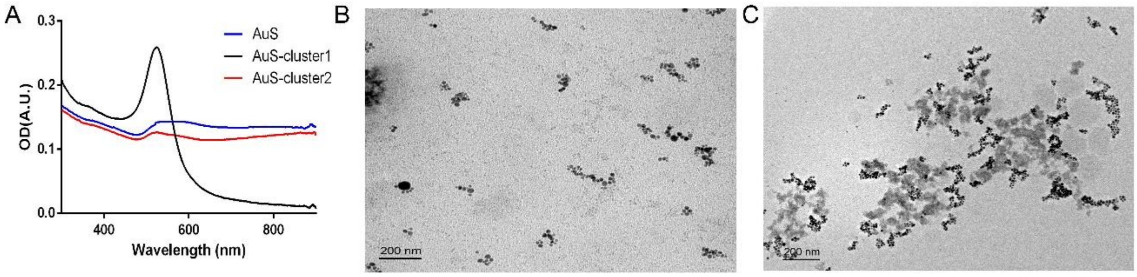

Figure S5. Properties of AuS cluster. Notes: (A)UV-vis absorption spectra of AuS and AuS cluster. (B, C) Transmission electron microscopy images of cluster 1 and cluster 2.
